# Dr. G. V. Black’s and Dr. J. Leon Williams’s Conclusions Reviewed

**Published:** 1897-12

**Authors:** James Truman


### DR. G. V. BLACK'S AND DR. J. LEON WILLIAMS'S CONCLUSIONS REVIEWED.[1]

BY JAMES TRUMAN, D.D.S.

The literature of dentistry has in the past two years been enriched by the investigations of two prominent workers, Drs. Black and Williams; for, however their conclusions may be criticised, there cannot be two opinions in regard to the value of their work. While this is conceded in the most generous terms, it is recognized that their deductions from their own labors are not satisfactory, and in some respects have had a very injurious effect upon minds who jump at conclusions on supposed authority, and these deductions are thus liable to become the prevailing thought of the dental profession. The statement that the work of these

[1] Read before The New York Institute of Stomatology, October 5, 1897.

industrious investigators is of great value seems hardly reconcilable with this view, but it must be remembered that original investigation leads to mental activity, and however erroneous the conclusions may appear to be, they induce to active thought and further work, and finally the truth is established. In this way all difficult problems have been solved, and it will continue, for generations to come, to be the open door for truth to enter.

Density, as generally understood, means "the mass or amount of matter per unit of bulk," or, as Newton gives it, "the property in bodies by which they contain a certain quantity of matter under a certain bulk or magnitude; closeness of contiguous parts; compactness." This is determined by specific gravity and by other tests. It has never been necessary for the practitioner of dentistry to be informed that some teeth represent a hardness equivalent to flint, and others are so soft that they are comparable to chalk, and have been thus named. According to Black, each of these presentations contain an equal amount of lime salts, or so nearly the same that the variation is not worth considering. In proof of this I quote Dr. Black's words (*Dental Cosmos*, May, 1893): "*These classifications, therefore, show conclusively that neither the density of the teeth nor the percentage of lime salts they contain has anything to do with the liability of the teeth to suffer from caries. In other words, the teeth of persons who suffer much from caries are just as hard, just as dense, just as heavy, and contain just as much lime salts as the teeth of persons who do not suffer from caries.*"

Now, this statement may be said to be true of teeth in general, but is very far from the truth when teeth are differentiated and examined in detail and in accordance with their apparent variations in density.

The difficulties surrounding an investigation of this character are practically insurmountable, for the simple reason that the material cannot be procured in sufficient quantity to make a test worthy of confidence. It counts for nothing if teeth be taken at random, and from this lot the decayed teeth be separated from the sound, and then the two lots be subjected to analysis after the Black method, with the result that each show equal quantities of lime salts. It would not require a very astute practitioner to decide this without investigation.

If, on the other hand, teeth had been selected from a mouth recognized as having chalky teeth, and these be compared with the aforementioned, and the result had been the same, it would not have been possible to have offered any criticisms, for even personal observations must give way to the crucible of analysis. Was this care exercised in Dr. Black's experiments? The answer to this may be expressed in his own language: "But a small number of the teeth used in this investigation have been extracted by myself. . . . In order to obtain material I arranged with certain gentlemen who extract many teeth to supply me. They were instructed to place the teeth in an envelope, just as they came from the mouth, write on the envelope the nativity, age, state of the general health, color of the hair, to class the teeth as bad, fair, or good, as to occurrence and rapidity of caries, record the condition of the gums, and mail the package to my address." Nothing in this, excepting "rapidity of caries," that would indicate that the extractor was to separate the so-called dense from the recognized chalky teeth. How any one extracting teeth indiscriminately could decide on rapidity of caries is a question not settled. Teeth are usually extracted because of continuous pain not amenable to treatment, or for the insertion of artificial teeth and treatment of irregularities. Caries is not confined to teeth of poor structure; indeed, it may be said, with some degree of truth, that this is no indication at all of the character or even the extent of the destruction, for all in long practice have met with very dense teeth melting down, as it were, under peculiar environments. It seems, therefore, amazing that so keen an observer as Dr. Black has proved himself to be should have based his whole work on such flimsy foundations, and makes this statement (p. 355) exceedingly fallacious: "The objects of the investigations here detailed, so far as they relate to the teeth, are to find the truth, if possible, as to the differences in the density, percentage of lime salts, strength, etc., occurring in the human teeth, and to find what relation, if any, such differences may bear to dental caries and other diseases of the teeth."

Then, again, we find him confining his investigations entirely to the dentine, for he says (p. 357), "I have chosen to confine the examination for density, proportion of water, lime salts, and organic matter to the dentine as best expressing the character of the tooth as a whole." While this may possibly have been the most convenient and best course to pursue, it leaves the coat of mail, the enamel, entirely out of the question, and this, the protective shield, is certainly an important factor in the inquiry.

Without extending the quotations from Dr. Black further, it may be said that if these deductions be accepted, then the observations in the practice of dentistry in the past decades must be valueless. It has been the observation from time immemorial that teeth do decay in proportion to the so-called density of the tissue. It has been universally recognized that teeth of soft structure yield to environments with a rapidity that defies the best work of the skilful dentist, while, on the other hand, the teeth regarded as dense resist to a great extent the effect of surroundings and give way slowly to destructive influences, oftentimes resisting to the extent of causing a cessation of caries. The reason for this latter result can be explained only theoretically upon the fact that some teeth are more dense than others. This has been the accepted hypothesis, and Dr. Black's experiments have failed, for the reasons given, to dissipate or weaken the supposition. Until the same careful examination be made in selected teeth, as heretofore described, this theory must stand. It will, however, be an extremely difficult task to make such an investigation, as the material is not to be procured. The teeth which are denominated chalky are usually found in young persons, and to remove these for the sake of investigation is entirely out of the question, hence evidence as to their relative density cannot be forthcoming.

If Dr. Black's conclusions are to be accepted, then it is certain that other factors besides the amount of lime salts have an important bearing upon their resisting power. What these are has not been determined, and until this is known we can safely leave the question where it has remained in practice, that there is a vital difference in teeth, and that caries is largely dependent upon structure.

I have given but a cursory view of Dr. Black's work in the present article, for the reason that his series of papers, as far as they relate to this subject, have been subjected to my criticism elsewhere. The *résumé* given has, therefore, been mainly to revive the matter in connection with the work of Dr. Williams.

Dr. Williams in his paper, continued in *Dental Cosmos* from March to May, 1897, has given so much that is extremely valuable that I have hesitated to take up any portion of the subject-matter in a critical spirit. The only inducement to do this is to be found in the expectation that perhaps we can reach the truth better by a frank discussion than by the method usually adopted, that of giving laudatory opinions, permitting the setting to obscure the supposed value of the gem enclosed.

The principal object of the paper read before the New York Odontological Society, January 12, 1897, was apparently to supplement Miller and Black's work by showing the effect of environment in producing dental caries.

The author has, practically, divided his work into two parts, the first being devoted to the effect of environment upon pits, depressions, and fissures in the teeth of animals, and the same in the human subject. He has covered much ground in this investigation, and apparently omitted nothing to demonstrate the truth as he understood it, and has presented the dental profession with pictures unexcelled heretofore, and it must be conceded has proved that the difference in the destruction between man and the lower forms is placed principally upon environment. Was the great labor devoted to this necessary? The fact has been recognized for generations, nay, more, proved, that given entirely natural conditions as to air, food, water, and surroundings, teeth will not be affected materially, and it has been equally well known that man has for uncounted years entirely ignored all of nature's laws in respect to eating and drinking, and has suffered in tooth deterioration and destruction.

There seems to be a wide distinction to be made between the fissures upon the occlusal surfaces of molars and bicuspids in man and that of the porcupine, a section of whose tooth is so beautifully represented. It must be apparent to the most careless observer that the defects in the formation of the enamel in the human teeth hold no relation to the perfect enamel surface of those illustrated, and this may be said of all the depressions of the various forms of carnivora, rodentia, ungulata, ruminantia, etc. It has been recognized, and requires no proof, that removal from these natural conditions means a change of environment and destruction. This has been evidenced in the rapid destruction of teeth of whole droves of pigs, as well as other animals, fed on still slops. No one, therefore, who has made but a superficial study of the effect of environment will question the position of Dr. Williams and Dr. Black upon this point, and it has been recognized as an undisputed question for a lengthened period. It, therefore, cannot be considered as anything very new or very important.

The following quotation is a marked evidence of attempting to antagonize a well-recognized fact by negative testimony: "A considerable proportion of abnormalities in human teeth have always been attributed to certain exanthematous diseases peculiar to childhood, but I have never heard it suggested that infant gorillas or youthful chimpanzees were liable to attacks of measles, chicken-pox, or scarlet fever, and so the many imperfections which I have discovered in the enamel of their teeth came as a great surprise to me." Is there any evidence that "infant gorillas or youthful chimpanzees" are not liable to diseases common to youthful humanity? The supposition would be equally as strong as his unsupported assertion that the exanthematous diseases may be, and of right ought to be, common with these forms, closely allied as they are to the human family. That these diseases do produce pits and other malformations in human teeth, through a disturbed nutrition, is as well proved as any problem in pathology, and nothing is gained by such assertions made under the guise of science.

The assertion is to my mind equally erroneous that, "Ask a dozen dentists, as you meet them, why human teeth are decaying so freely and rapidly to-day while the teeth of animals are not, and a majority of them will answer, Because the structure of human teeth is not so good as that found in the teeth of animals." This must be regarded as a pure creation of the imagination, as no one at all intelligent upon the subject would make such a statement.

Dr. Williams endorses very fully Dr. Black's work, and states that "notwithstanding the conclusions reached by Dr. Black, the opinion is still generally held by the dental profession that decay of the teeth is primarily due to degeneration which has taken place in the tissues of these organs." Degeneration does not seem to be the proper word to use, if it is intended to imply that a change takes place in the structure of the teeth during the lifetime of the individual. No one will, it is presumed, take this untenable position. If, however, is meant degeneration through successive generations, then the profession holds to the correct view. The possibility of a loss of vital power through changing environments and continued local systemic disturbances is properly recognized as prominent factors in deterioration. These are temporary disturbing elements to be corrected through time and improved conditions. The effect of climate, food, and deleterious surroundings produce changes well understood, and these may continue for generations, to be eventually succeeded by a gradual return to original types, for this seems to be an invariable law of nature; but this is not confined to the teeth, but applies with equal force to every organ of the body.

It is questionable whether this statement of Dr. Williams is absolutely correct, if his meaning be understood, that the opinion is generally held by the dental profession that decay of the teeth is primarily due to degeneration, but it is certainly true that many capable of truly measuring facts have decided otherwise. Talbot ("Etiology of Osseous Deformities") says that "certain monogenists have held that a human race could not effect a change of extreme climate without the loss of life, while others maintained exactly opposite opinions. At any rate, we know that many races have passed these two extremes, but it causes an entire change in the structure of the offspring, both in man and beast as well as fruit and vegetables. Every race being a resultant whose components are partly the species itself, partly the sum of the modifying agencies, has produced deviations from the original stock." When, therefore, Dr. Williams repudiates the possibility of degeneration of teeth he sets aside facts as well established as the process of development of teeth.

In dismissing this part of Dr. Williams's work it must gratefully be acknowledged that he has given the dental world the most perfect illustrations, in discussing this subject, that have, probably, ever been seen, and these will remain as a perpetual evidence of the partial truth that decay is the product of environment, but they do not confirm this theory to the exclusion of that other, that structure is equally an important factor in destruction. In a word, it nowhere sustains Dr. Black's contention, if I am capable, through long and careful study of the paper, of understanding and appreciating his conclusions.

The portion of Dr. Williams's work devoted to antagonizing views held by Abbott, Heitzmann, and Bödecker, may be left as he places it, but it must be conceded that his demonstrations fall very far short of proving the correctness of his theory that sensation of enamel is not compatible with his demonstrations. His illustrations disproving the fibre theory of these microscopists may or may not be conclusive, but he does show that it is not difficult to make errors of observation through imperfect microscopical manipulation, a fact well understood by all workers in that direction.

The fact that sensation does exist in enamel cannot successfully be disputed. It, probably, has been recognized by every practitioner, and in exceptional cases is equal in intensity to that of dentine. Theoretically this should be existent, in moderate degree, for how otherwise would it be possible to understand the tactile sensibility of the enamel which Kölliker sought to explain by the action of the molecules of matter acting upon each other and eventually upon the pulp?

The definition which Williams gives to the term organic seems unsupported by evidence. An organ is understood as a "part of an organized body, which has some specific function." "The term organic as applied to any substance in no way relates to the presence or absence of life." On page 281, Fig. 45, is represented a "specimen of nearly decalcified enamel. . . . This specimen was mounted in glycerol, and pressure upon the cover glass showed that the portion outside of the line of striation moved slightly, but was held to the main mass by delicate fibres, which were not *between* the rods, but which *constituted the organic basis of the rods themselves.*" (Italics mine.) In thus stating, he concedes that there is an organic basis for enamel as well as of dentine. This means, in my opinion, possibilities not, seemingly, recognized by Williams. We are still left to conjecture as to the function of this organic matter, whether it constitutes a fixed proportion of the enamel, as the Heitzmann school held, or remained in very minute quantities, as Tomes sought to demonstrate. We know nothing as to the function of the sheaths of Neumann, but the fact that they are, practically, indestructible to acids proves that they are not inorganic, whatever else they may be, and the analogues to these in enamel may be, and probably are, part of the formative tissue, and suggest the possibility of these being the medium for the tactile sensibility of the teeth.

This point, while interesting as a subject for investigation, does not concern us at present. The main question to be considered, as I view it, is, Have we learned anything from Dr. Williams's exhibit in regard to caries? The answer must come to this from his own work. He evidently regards the results obtained as supplementary to that of Miller and Black, and that it, practically, completes the investigations into the origin of dental caries, in that it shows the beginning of dissolution of the enamel border, and eventual destruction of that tissue to which but slight attention has been given, either by the observers named or by those preceding them.

It is to the very great credit of Dr. Williams that he has taken up this difficult subject, for if all that he claims cannot be conceded, he has given actual demonstrations of the origin of decay in enamel in a way never before successfully attempted, owing to difficulties in technique heretofore supposed insurmountable.

While this is recognized, the deductions drawn from the illustrations cannot be accepted. When Dr. Williams asserts that the appearances indicate "*that decay was the result of some specific cause acting continuously at some particular point in a manner impossible to free acids in the mouth,*" he assumes a proposition that he does not prove. This is made stronger when further on he states, "*This mass of fungi is so dense and adhesive as to make it highly improbable that the enamel is affected, except in rare and special instances, by any*

*acid other than that which is being excreted by the bacteria at the very point where they are attached to the enamel.*" It is possible that this supposition may be well grounded, but the author gives no evidence that enamel is destroyed through the acid excreted by the organisms, and such evidence has never been given with sufficient accuracy to make it worthy of acceptation. The mere fact that destruction is shown to have taken place beneath the fungi depicted has no force.

It may be well to consider what these forms really represent. They are originally known as leptothrix buccalis, the name given by Robin, 1847, and previously given various names, as Bühlmann's fibres, denticolæ, etc. Miller devotes considerable space to their consideration in his work on the "Micro-Organisms of the Human Mouth," and says, "As absolutely nothing was known concerning the biology and pathogenesis of this organism, all sorts of wonderful properties were ascribed to it." He divides these under several names,—"leptothrix innominata, bacillus buccalis maximus, and leptothrix buccalis maxima,"—but nowhere does he give these forms credit for the immediate destruction through the production of acid; in fact, has never been able to effect a pure culture out of the mouth. The assertion of Dr. Jung (Translation, INTERNATIONAL DENTAL JOURNAL, March, 1897), that "the leptothrix appears to play only a secondary part in caries according to present ideas," may be regarded as the most recent utterance from that authority, as Jung has been very close to Dr. Miller as his assistant. The theory of Leber and Rottenstein that leptothrix entered the tubulated structure after acid action is the nearest approach to the theory of Williams that I am familiar with, if we except Vignal, whose work with these organisms has not been corroborated.[1] The fact that these forms have been recognized as taking some part in caries is nothing new. It was taught fully a quarter of a century ago, but the *rationale* of the process was as much in the dark then as now, and the work of Miller finally placed a quietus upon the idea until its revival, upon insufficiant data, at the present time.

It is possible, indeed it is highly probable, that this mat of

---

[1] Since this review was written, a paper has appeared in the October number of the *Journal of the British Dental Association*, by T. Choquet, D.E.D.P., Paris, on the Leptothrix Buccalis. He claims that this fungi is a "peculiar species," and thus endorses Vignal's work. He also confirms Miller's idea that several distinct forms of Leptothrix exist. Choquet was not able to demonstrate their pathological character. This interesting paper will be found under the head of Abstracts and Translations. J. T.

fungi has an effect, but not, as far as known, through the method suggested. The beautiful illustrations show clearly acid action, but is this necessarily the product of fungi?

The collection of so-called leptothrix in all slow-progressing caries forms a mat of considerable thickness over the dentine, and may be found on enamel and in other portions of the mouth. In a cavity, or externally, it unquestionably furnishes a retention place for the acids of fermentation, and as leptothrix seems to thrive best in an acid fluid, it holds the destructive agent in close juxtaposition to the dentine and enamel present, and dissolution of these rapidly supervenes. The same effect is produced and, in my opinion, in the same way by the introduction of cotton in a cavity. This will produce extreme hyperæsthesia in a few hours. Until Dr. Williams succeeds in proving that the fungi he shows in connection with enamel is acid-producing, his theory will fail to convince those at all familiar with the subject that this is the origin of caries through direct action.

Fermentation in the mouth cannot be set aside as a direct producer of destruction of the tissues of the teeth. This in no way invalidates the work of Miller, for no one has more thorough confidence in his conclusions than myself. The origin of caries, in its larger aspect, I regard as having been settled through his thorough and exhaustive work, but while conceding this I am not prepared to believe that his conclusions cover all the destruction witnessed in the oral cavity, nor would it be proper to dogmatically oppose these merely upon operations in practice or possibly crude microscopic examinations.

Some years ago I called attention to the fact that in investigating the origin of erosion I found that it was an error to confine the tests to the period when the alkaline saliva was most active in neutralizing the formative processes, but that it must be continued to periods when all the functions of the body were in comparative rest; in other words, at night. The result of my own observations and that of others, following my own line of work, confirmed the fact that fermentation was very active at this period. Tests in sufficient number of cases demonstrated that the fluids of the mouth gave neutral response during the day, and that so continually that this was decided to be the normal reaction during the period of greatest activity. The reaction at night was invariably acid. This led to the conclusion that all conditions of rest would produce a similar result, and this was proved to be correct by tests in cavities remote from the action of the saliva.

Accepting this as a demonstrated fact, what, then, may we conclude, that the acids produced by fermentation will destroy the enamel, as in erosion, without the direct action of micro-organisms? This, without disturbing accepted theories, simply points to other factors as taking part in the destruction of teeth.

Multiplication of words is not required to demonstrate to the intelligent practitioner that he constantly meets with teeth that are far below the average of normal structure. This soft, chalky condition is an existent quality, and no amount of investigation, after the methods of Black, will convince to the contrary. These are the teeth that are directly affected by what Black and Williams call the environments, and this is perhaps the correct term to use. The acid action upon this character of tooth-structure is too rapid to indicate micro-organic co-operation. It is direct chemical action, breaking down the inorganic elements and leaving the organic, or basis substance of the dentine, for this resists the disintegrating force for a longer period. Microscopic examination of portions of this tissue has shown that the organic base is destroyed more rapidly than the sheaths of Neumann, these being left prominently extending beyond the mass. This, from their indestructible nature, should be expected. Whether the protoplasmic fibre of Tomes remains intact in these sheaths cannot be demonstrated, but the great increase of sensitiveness in that class of teeth would indicate that they remain longer undisturbed than the inorganic portion and are subject to constant irritation through the presence of acids.

The conclusions I have reached from a study of the entire subject may be summed up as follows:

1. That Black succeeded in demonstrating that there was practically no difference in density in the teeth he subjected to examination and failed, in that he classed all teeth with caries as representing all variety of structure, and this being an error, his conclusions to that extent are weakened.

2. Dr. Williams in his attempt to demonstrate that there was no difference between human and animal teeth failed to consider of sufficient importance the unnatural conditions and changing environment not present in inferior forms.

3. It is not proved that micro-organisms have any direct surface influence on enamel, as Dr. Williams asserts that they have.

4. The cause of caries remains as Miller left it, if the subject be studied from comparatively dense teeth; but where the structure is below normal the factor of environment takes a pre-eminent position; in other words, direct chemical action supersedes, if it does not altogether take the place of, the intermediate action of micro-organisms.

If my conclusions be correct, the destruction of teeth can only be properly accounted for upon the facts established by Miller, combined with the older chemical theory of which Magitot was the leading exponent.

---

